# Biomechanical Analysis of Different K-wire Configurations for Percutaneous Fixation of Two-Part Proximal Humerus Fractures

**DOI:** 10.7759/cureus.73848

**Published:** 2024-11-17

**Authors:** Haidar Nasuruddin, Mohd Azfan Che Yusoff, Aminudin Che Ahmad, Muhammad Harith Rosdi

**Affiliations:** 1 Orthopedics, Traumatology and Rehabilitation, International Islamic University Malaysia, Kuantan, MYS

**Keywords:** biomechanics, kirschner wire (k-wire), osteoporotic fractures, percutaneous fixation, proximal humerus fractures

## Abstract

The increasing prevalence of proximal humerus fractures in the elderly population, particularly osteoporotic fractures, necessitates a biomechanical evaluation of Kirschner wire (K-wire) configurations used in percutaneous fixation. This study investigates the stability of different K-wire configurations and examines the effect of wire size and type (smooth vs. threaded). Using 27 synthetic humeri models, we compare three configurations as follows: four parallel ascending K-wires (box-type), two ascending and two descending K-wires, and a combination of both. Results show that adding descending K-wires significantly enhances stability, particularly against torsional forces. This study highlights the importance of wire type and configuration in stabilizing proximal humerus fractures. Multiplanar constructs with descending K-wires, especially threaded ones, offer better stability. These insights help improve surgical techniques for elderly and osteoporotic patients, but further research using cadaver models is needed for clinical validation.

## Introduction

Proximal humerus fractures, particularly common in the elderly population, present a significant clinical challenge due to their association with osteoporosis and the complexity of bone anatomy in this region [1,2]. The humeral head and surrounding tuberosities are subject to biomechanical forces that complicate fracture fixation, with a growing need to optimize surgical techniques for improved patient outcomes. With the global rise in osteoporosis and aging populations, the incidence of such fractures is projected to increase, emphasizing the importance of effective and stable surgical interventions [3,4].

Several surgical methods have been developed for proximal humerus fractures, including percutaneous pinning, intramedullary nailing, and open reduction with plate fixation. Among these, percutaneous pinning is often preferred for its minimally invasive nature, which reduces soft tissue disruption and preserves blood supply. However, complications such as pin migration and mechanical instability have prompted ongoing research into the optimal configuration and material of K-wires used in these procedures [5,6].

This study aimed to investigate the biomechanical stability of various K-wire configurations and sizes in the fixation of two-part proximal humerus fractures. Using synthetic humeri models, we examine the effects of wire size, material type, and pin arrangement on torsional stability, pin migration, and mechanical resistance to further refine clinical recommendations for optimal fracture management.

## Materials and methods

This experimental study was conducted using synthetic left humerus models from SYNBONE (N=27), which were chosen for their anatomical consistency and reliable mechanical properties. Previous studies have validated synthetic bones as a substitute for cadaveric bones in biomechanical experiments, citing similar fracture resistance and behavior under mechanical stress [6,7]. Each synthetic humerus underwent a transverse osteotomy at the surgical neck 35 mm below the greater tuberosity, stimulating a two-part proximal humerus fracture.

**Figure 1:**
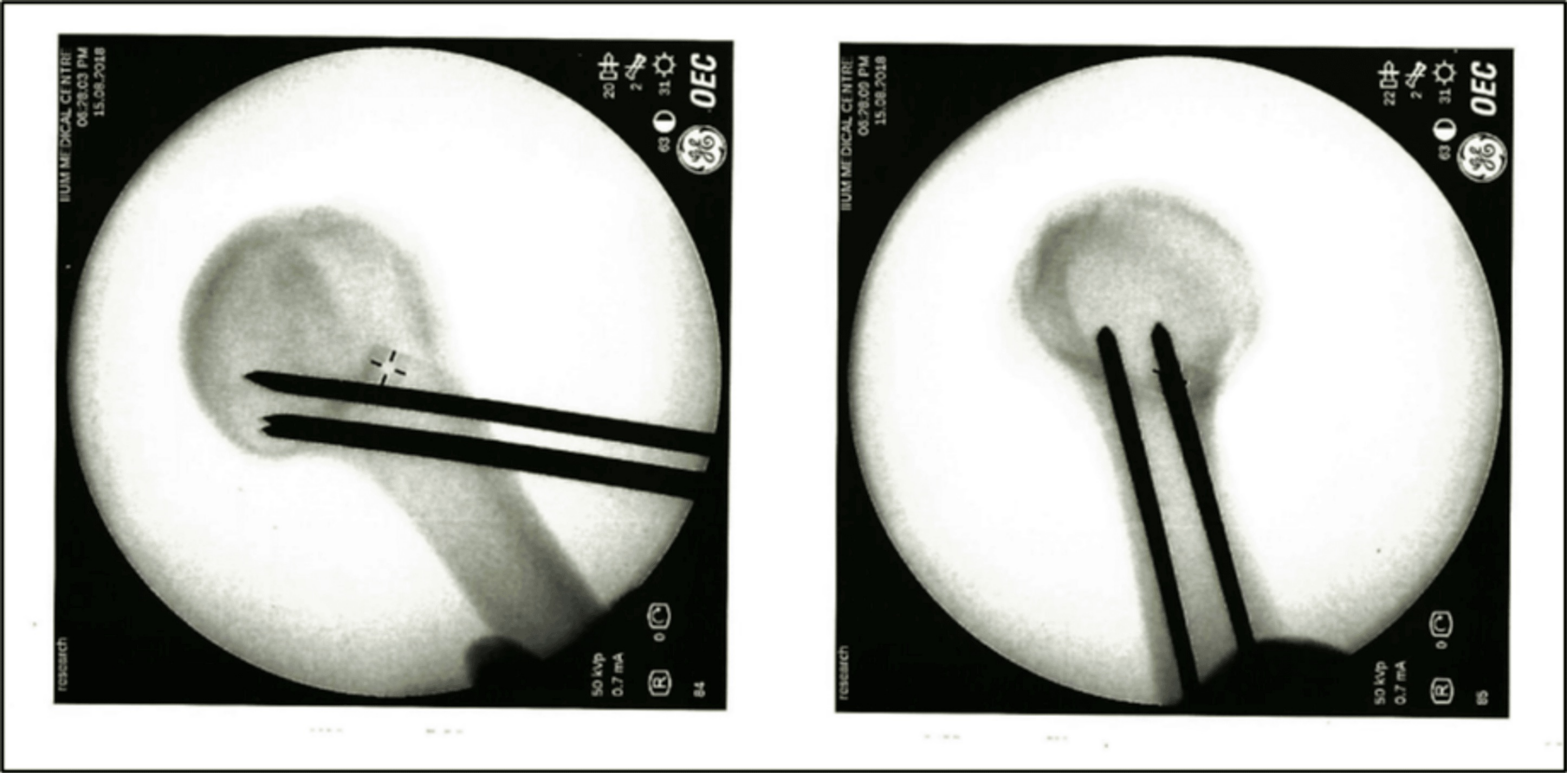
Group 1 - four ascending parallel wires.

**Figure 2:**
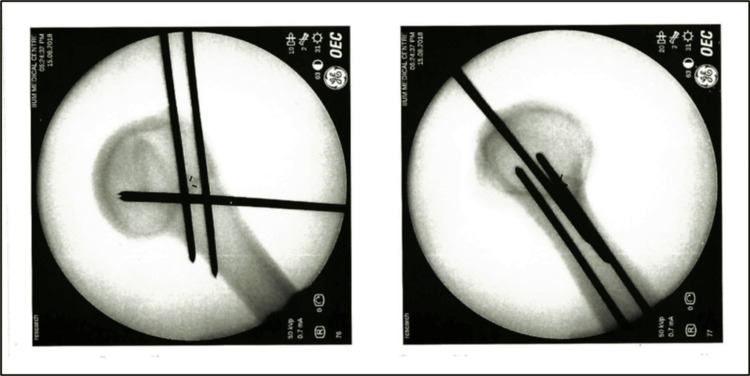
Group 2 - two ascending and two descending wires.

**Figure 3:**
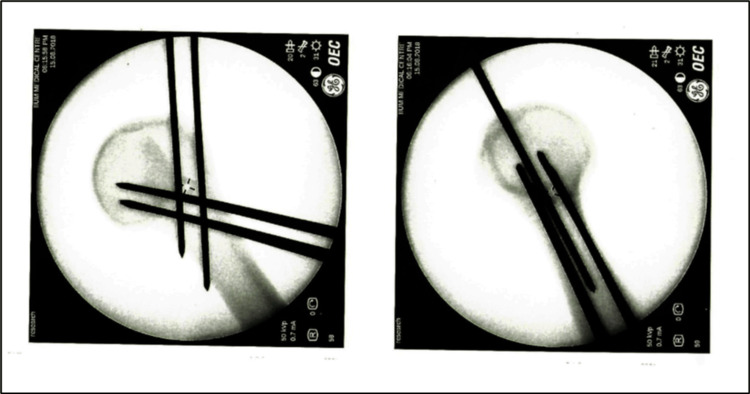
Group 3 - four ascending and two descending wires.

Three distinct K-wire configurations were tested across different groups. In Group 1, the configuration consisted of four parallel ascending K-wires arranged in a box-type formation (Figure 1). Group 2 featured a setup with two parallel ascending K-wires combined with two descending K-wires (Figure 2). Meanwhile, Group 3 incorporated four parallel ascending K-wires, which were further complemented by two descending K-wires inserted from the greater tuberosity (Figure 3). These varied configurations allowed for a comparative analysis of the effectiveness and stability provided by each approach.

In this study, various configurations were applied to different types of K-wires based on their diameter and surface finish. The wires were divided into three distinct subgroups. Subgroup A consisted of smooth K-wires with a diameter of 2.0 mm, while subgroup B involved smooth K-wires of a slightly larger diameter, measuring 2.5 mm. Lastly, subgroup C included 2.5 mm threaded K-wires, which differ from the smooth varieties by having a helical surface that enhances bone grip. These distinctions in diameter and surface finish allowed for a comparative analysis of the performance characteristics of each type under different configurations.

The wires were inserted by a single operator, and their placement was verified through fluoroscopy. Threaded wires were tested to evaluate their effect on biomechanical stability, following evidence that threaded surfaces may enhance cortical bone grip [8].

Biomechanical testing on rotational stability

The primary measure of biomechanical performance was torsional stiffness, quantified using a torsion testing machine. The synthetic humeral head was placed in a fixed cylindrical jig, and a rotational force was applied until 10° of displacement was achieved. The torque required to achieve this displacement was recorded as a measure of construct stiffness.

The secondary outcome was the incidence of pin migration and loosening, which was visually inspected after mechanical testing. These complications were noted as important clinical challenges in earlier studies [5,9-11].

Sample size calculation

The minimum sample size was determined for each of the study objectives, whereby the largest sample size was generated using the following formula:

$n=\frac{(Z_{\frac{a}{2}})^{2}&times;p&times;q}{d^{2}}$

Here, $Z_{\frac{a}{2}}$=1.96, estimated proportion (p) = 93.5%, q = 6.5%, and desired absolute precision (d) = 10% [12]. With the anticipation of a 10% non-response rate, the total number of respondents required was 27.

Statistical analysis

All analyses were conducted using IBM SPSS Statistics for Windows, version 24.0, released in 2016 (Armonk, NY: IBM Corp.). Data were analyzed using descriptive statistics to summarize the outcomes for each group. The Kruskal-Wallis test was employed to compare differences between the three wire configurations, followed by post hoc pairwise comparisons using the Mann-Whitney U test. A p-value of less than 0.05 was considered statistically significant.

## Results

All 27 specimens successfully withstood the testing procedures without any failures or deformation of the K-wires. Torque measurements (in Nm) at 10° displacement ranged from 0.25 Nm to 0.65 Nm. A comparison of the median stiffness values for all groups and subgroups is presented in Table 1.

**Table 1:** Distribution of stiffness values for all groups and subgroups.

Variables	Group	1			2			3		
	Subgroup	A	B	C	A	B	C	A	B	C
Torque at 10° (Nm)	Median	0.50	0.45	0.30	0.45	0.50	0.55	0.55	0.55	0.55
	Minimum	0.45	0.45	0.25	0.40	0.40	0.55	0.40	0.50	0.55
	Maximum	0.50	0.50	0.35	0.50	0.55	0.55	0.60	0.65	0.55

In Table 2, in subgroup C (2.5 mm threaded wires), Group 1, which utilized four ascending parallel K-wires, exhibited statistically significantly lower stiffness compared to Groups 2 and 3 with a p-value obtained of 0.022 (p<0.05). Although Group 1 also demonstrated lower stiffness in subgroup B, the differences were not statistically significant (p=0.197). A similar outcome was for subgroup A, where no statistical significance was obtained between Groups 1 and 3 (p=0.513).

**Table 2:** Kruskal-Wallis test on K-wire types. *P<0.05 was considered significant. df: degree of freedom

Type of K-wire	Torque at 10° (Nm)	Frequency (N)	Median	Mean rank	Chi-square	df	p-Value
A	1	3	0.50	5.17	1.33	2	0.513
	2	3	0.45	3.67			
	3	3	0.55	6.17			
B	1	3	0.45	3.33	3.25	2	0.197
	2	3	0.50	4.50			
	3	3	0.55	7.17			
C	1	3	0.30	2.00	7.62	2	0.022*
	2	3	0.55	6.50			
	3	3	0.55	6.50			

The post hoc test using the Mann-Whitney U test concluded that there was no statistically significant difference between each pair, between subgroups A and B, subgroup A and C, and subgroup B and C, when all the p-values were recorded higher than 0.05 (Table 3).

**Table 3:** Mann-Whitney U test among K-wire types. *P<0.05 was considered significant. df: degree of freedom

Variables	Type of K-wire	Frequency (N)	Median	1st quartile	3rd quartile	Mean rank	Sum of ranks	Mann-Whitney U	p-Value
Torque at 10° (Nm)	A	9	0.50	0.43	0.53	8.72	78.50	33.50	0.525
	B	9	0.50	0.45	0.55	10.28	92.50		
	A	9	0.50	0.43	0.53	9.00	81.00	36.00	0.681
	C	9	0.55	0.33	0.55	10.00	90.00		
	B	9	0.50	0.45	0.55	9.33	84.00	39.00	0.889
	C	9	0.55	0.33	0.55	9.67	87.00		

## Discussion

Proximal humerus fractures represent approximately half of all humerus fractures, frequently affecting the elderly and predominantly women over 70 years of age. As the global population continues to age, the incidence of these fractures is projected to increase substantially [1]. These fractures, primarily osteoporotic in origin, commonly occur from low-energy trauma, such as falls, though high-energy trauma like vehicular accidents is often the cause in younger patients [2]. Managing fractures in elderly patients with comorbidities, including diabetes, presents distinct challenges because of increased risks of surgical complications and infections. Proximal humerus fractures vary in severity, ranging from stable fractures, such as Neer Type 1, to complex multipart fractures. While conservative treatment suffices for stable, undisplaced fractures, which make up 40% of cases, surgical intervention is often required for more complex fractures [3]. Given the bone fragility in elderly populations, two-part fractures at the surgical neck are increasingly common, with cases of three-part or four-part fractures also becoming prevalent.

In cases of displaced fractures, surgery becomes necessary to achieve stable fixation. One prominent surgical approach is percutaneous pinning, first introduced by Böhler in 1964 [13]. This minimally invasive technique has demonstrated positive outcomes in elderly patients, as it preserves blood supply and limits surgical exposure, minimizing risks of avascular necrosis [5,11]. Percutaneous pinning also offers benefits in terms of reduced scarring and joint stiffness compared to open reduction and internal fixation (ORIF) [12,14]. However, despite these benefits, percutaneous pinning is associated with complications, such as pin migration, loosening, infection, and potential fixation failure [9]. While pinning is widely used for treating simple fractures, it provides limited stability in cases involving varus collapse or calcar comminution, and it is therefore contraindicated in patients with severely comminuted fractures or osteopenic bones.

This study focused on identifying the optimal K-wire configuration for achieving stable fixation in two-part proximal humerus fractures. The use of K-wires, especially with the J-nailing technique, demonstrates effectiveness, with a reported mean union time of approximately 13.5 weeks [15]. Biomechanical analysis has shown that K-wire positioning and angulation are critical to enhancing stability and reducing interfragmentary strain, promoting effective healing [16]. In this study, three K-wire configurations were tested on synthetic humeri, which offer consistent size and mechanical properties, thus providing a suitable alternative to cadaveric models [7,17]. Group 1 utilized four parallel ascending wires, a configuration previously shown to provide stability [7]. Group 2 incorporated two descending wires along with two ascending wires, as described by Naidu et al., while Group 3 combined elements from both configurations [6].

The findings indicated that configurations involving descending wires (Groups 2 and 3) enhanced stability significantly more than configurations with only ascending wires. The descending wires contributed to stability by engaging the medial cortex, which increased resistance to varus and rotational forces that are crucial for stable fixation [14]. These findings are consistent with prior research, such as by Durigan Jr et al., which found that two lateral ascending wires combined with two descending wires from the greater tuberosity were highly effective in resisting varus stresses [14]. The current study further validated that descending wires improve rotational stability, a critical factor for maintaining secure fixation.

Jiang et al. also explored the use of four parallel ascending pins in comparison to four converging pins in cadaveric models and found that parallel ascending pins provided greater torsional stability [7]. This finding aligns with the current study’s results and supports the decision to focus on parallel configurations. Moreover, this study evaluated the effects of different wire diameters (2.0 mm vs. 2.5 mm) and wire ends (smooth vs. threaded). Although no significant differences in stability were observed between wire sizes or types, threaded wires are suggested to offer clinical advantages due to their superior holding power, which reduces risks of migration and loosening [5]. Herscovici Jr et al. also reported that smooth K-wires were associated with a higher incidence of pin loosening, reinforcing the clinical preference for threaded wires [5].

Percutaneous pinning with K-wire fixation was also compared to ORIF using locking plates, such as the Proximal Humerus Internal Locking System (PHILOS) plate, to assess clinical outcomes. Studies indicate that both K-wire fixation and PHILOS can provide satisfactory results, although PHILOS generally offers more secure fixation [18,19]. In one cohort study, favorable outcomes were achieved in 72% of K-wire cases, while PHILOS reached an 88% success rate [19]. While K-wire fixation has significant advantages regarding soft tissue preservation, it may not match the stability provided by locking plates, particularly in more complex fractures. Ongoing research aimed to refine K-wire configurations to improve outcomes further, particularly in elderly populations where preserving soft tissue is crucial.

Based on the findings, the most stable percutaneous fixation for two-part proximal humerus fractures appears to involve a combination of ascending lateral wires and descending wires from the greater tuberosity. This configuration enhances stability by engaging the medial cortex, thereby increasing resistance to varus and rotational forces. Despite these advantages, there are anatomical risks associated with the placement of wires near critical structures, such as the axillary nerve and posterior circumflex humeral artery. Careful surgical technique is essential to avoid neurovascular complications, particularly when inserting wires through the greater tuberosity [15].

In summary, percutaneous pinning with K-wires remains a viable option for treating two-part proximal humerus fractures, particularly in elderly patients with osteoporosis. Configurations involving descending wires have demonstrated superior stability and resistance to rotational forces, making them favorable for managing these fractures. However, further research is needed to refine K-wire techniques, assess their efficacy in complex fracture patterns, and evaluate long-term clinical outcomes associated with these configurations. The benefits of minimally invasive techniques like percutaneous pinning are substantial, particularly for older patients, underscoring the importance of optimizing these approaches to balance stability with the preservation of soft tissue and vascular integrity.

Clinical implications

The findings of this study have direct applications in the surgical management of proximal humerus fractures, particularly for elderly patients. Surgeons should consider using threaded K-wires and a multiplanar configuration (as seen in Group 3) to improve fixation stability, particularly in osteoporotic patients. The biomechanical performance of these configurations suggests that they offer better resistance to torsional and bending forces, reducing the risk of malunion and non-union.

Furthermore, the use of synthetic humeri in this study validates their utility in biomechanical testing, offering a consistent and reproducible model that can be used to evaluate different surgical techniques and materials. However, the limitations of synthetic bones, including their inability to fully replicate human bone properties, should be considered when interpreting these findings.

Limitations

The primary limitation of this study is the use of synthetic bones, which, while offering consistent anatomical properties, do not fully replicate the biological and mechanical characteristics of human bone. The role of soft tissue in stabilizing and counteracting the deforming forces was not included. Additionally, this study only evaluated torsional stability, whereas other forces (such as bending and axial loading) are also relevant in vivo. Future studies should expand on this by using cadaveric models and testing under more varied loading conditions.

## Conclusions

This study underscores the importance of wire configuration and type in achieving stable fixation for proximal humerus fractures. The use of multiplanar constructs with descending K-wires provides superior mechanical stability, particularly when threaded wires are used. These findings offer valuable insights into optimizing surgical techniques for elderly and osteoporotic patients, where achieving stable fixation is often more challenging. Further research, particularly involving cadaveric models, is necessary to confirm these findings under more clinically relevant conditions.
